# The corpus callosum in Binswanger’s disease: A quantitative fractional anisotropy analysis

**DOI:** 10.1590/S1980-57642009DN20400008

**Published:** 2008

**Authors:** Eliasz Engelhardt, Denise Madeira Moreira, Gilberto Oliveira Alves, Maria Elisa Oliveira Lanna, Carlos Eduardo de Oliveira Alves, Letice Ericeira-Valente, Felipe Kenji Sudo, Jerson Laks

**Affiliations:** 1-10 Center for Alzheimer’s Disease / Institute of Psychiatry/Federal University of Rio de Janeiro; School of Medical Sciences, State University of Rio de Janeiro. Institute of Neurology Deolindo Couto of the Federal University of Rio de Janeiro.

## Abstract

To study the integrity of the corpus callosum in Binswanger’s disease (BD) patients using quantitative fractional anisotropy (DTI-FA).

**Methods:**

Controls (12) and patients with BD (12) were included. MR [GE Signa Horizon-1.5T] scans were performed. BD patients presented Fazekas’s score=6 and leukoaraiosis extension =75%, as assessed on FLAIR sequence. Standard parameters for DTI-FA acquisition were used. Functool was employed for post-processing, and ROIs placed on the genu and splenium of the corpus callosum on one axial plane at the basal ganglia level. Statistics [ANOVA] for genu and splenium comparison were analyzed.

**Results:**

DTI-FA showed reduction of anisotropy in both regions of the corpus callosum, more prominently in anterior (genu) than posterior (splenium) in BD patients versus controls.

**Conclusion:**

The reduction of anisotropy reflects loss of integrity of fibers of the studied regions of the corpus callosum. This finding indicates an interruption of the most important inter-hemispheric commissure, and component of neural networks that underlies cognitive, behavioral, motor and sensory integration. The affected genu and splenium, together with damage to other fiber systems that connect the prefrontal and parietal-occipital regions, may manifest clinically as dysfunction of high-level integrative regions linked to the domains of executive and sensory functions, respectively, that can occur in Binswanger’s disease.

## Abstract (pt)

Estudar a integridade do corpo caloso em pacientes com doença de Binswanger (DB) com anisotropia fracionada quantitativa (DTI-FA).

**Métodos:**

Foram incluídos controles (12) e pacientes com DB (12). Imagens de RM [GE Signa Horizon-1,5T] foram obtidas. Os pacientes com DB apresentavam escore=6 de Fazekas e leucoaraiose com extensão =75% como avaliado na seqüência em FLAIR. Foram utilizados parâmetros padrão para DTI-FA e utilizado Functool para pós-processamento. ROIs localizados no joelho e no esplênio do corpo caloso em um plano axial no nível dos gânglios da base. Estatística [ANOVA] feita para comparar joelho e esplênio.

**Resultados:**

DTI-FA mostrou redução da anisotropia em ambas as regiões do corpo caloso, com predomínio na anterior (joelho) em relação à posterior (esplênio), nos pacientes com DB em comparação aos controles.

**Conclusões:**

A redução da anisotropia reflete perda da integridade de fibras das regiões estudadas do corpo caloso. Esses achados indicam interrupção da mais importante comissura inter-hemisférica e componente de redes neurais subjacentes à integração cognitiva, comportamental e de funções motora e sensorial. O comprometimento do joelho e do esplênio, juntamente com a lesão de outros sistemas de fibras, que conectam regiões pré-frontais e parieto-occipitais, podem ser expressas clinicamente por disfunção de regiões de alto nível de integração, relacionadas aos domínios das funções executiva e sensorial, respectivamente, que pode ocorrer na doença de Binswanger.

The corpus callosum is the largest fiber tract, and the main commissure, of the human brain, interconnecting neocortical areas of both hemispheres. The free part (midline) of the corpus callosum is easily visible (better seen in midsagital cuts), and includes a medial and a lateral part (defined as the region adjoining the callosal recess). From here the fibers spread out as the radiations of the corpus callosum that merge into the hemispheric white matter where they intermingle with other tracts in the centrum semiovale and with the fibers of the corona radiata. It may be divided into roughly three regions, anterior (incorporating the genum), intermediary (body), and posterior (incorporating the splenium). The genu connects the prefrontal regions, the body connects the posterior portion of the frontal lobes and the parietal lobes, while the splenium connects regions of the parietal and occipital lobes.^1-4^

The corpus callosum establishes inter-hemispheric connections in a topographically organized way, and functionally information transfer takes place between areas related to cognition, behavior, motor and sensory functions.^2,5-9^

This commissure is provided with a rich arterial supply through the pericallosal, posterior pericallosal, and the anterior communicating arteries. They form the pericallosal pial plexus that supplies the midline corpus callosum and part of its radiations. The midline of the corpus callosum also presents a specific microvascular supply that boosts its vascularization. The vascular supply to the central zone of the genu and body of the corpus callosum, via short penetrating arterioles, is similar to that of the cerebral cortex, whereas the vascular supply to the extreme lateral corpus callosum and centrum semiovale is largely carried by medullary arteries, long end-arteries without anastomoses.^4,10-12^

The corpus callosum may be affected by numerous diseases, including those of vascular etiology. Infarctions and atrophy as a consequence of widespread subcortical white matter ischemic diseases, such as Binswanger’s disease, may also occur.^3,13^

Binswanger’s disease (BD) is one of several subtypes of the vascular cognitive impairment-vascular dementia (VCI-VaD) *continuum*.^14-15^ Pathological examination of BD brains reveal atrophy, and the cut sections show an enlarged ventricular system, hypotrophy and yellowish discoloration of the subcortical white matter, and a thinned corpus callosum.^16^ The thinning of the corpus callosum is often secondary, and primary white matter lesions have not been reported in this region.^3,17^ The microscopic neuropathology of white matter lesions is mainly characterized by diffuse loss of nerve fibers, demyelination and gliosis affecting the centrum semiovale and the corpus callosum in a differentiated way.^3,16,18-20^ The lateral part of the corpus callosum may be affected secondary to deep white matter lesions, while its medial part is “rarely affected or only affected in a non significant manner”.^3^

The large brain vessels in BD show atherosclerotic changes, and the histopathology reveals microvascular disease in the form of severe arteriolar sclerosis, especially in subcortical white matter.^16,10^ This small-artery pathology is likely to be one of the underlying substract of the extensive white matter lesions, and is frequently related to hypertension, diabetes mellitus, dyslipidemia, and other vascular risk factors.^19,21-24^ However, vascular changes commonly seen in the centrum semiovale with aging or hypertension rarely develop in the corpus callosum, probably due to its special vascular supply which may explain its relative resistance to lacunar infarction, hypoxia, hypoperfusion, and Binswanger’s disease.^22^

Structural imaging techniques (computer tomography and magnetic resonance) and the concept of leukoaraiosis that followed^20,24-28^ allowed the observation that BD had a much higher prevalence than formerly thought, and provided the opportunity to establish the diagnosis *in vivo*.^19,29^

These conventional neuroimaging methods showed wide confluent areas of white matter disease in cases of Binswanger’s disease, identified neuropathologically as being of ischemic cause.^19,20,22,24,26,28^, However, such findings were rarely described in the corpus callosum.^3,17^

The recently developed technique of diffusion tensor imaging (DTI) has offered a new opportunity to evaluate the brain white matter architecture in a qualitative and quantitative way, in both normal and pathological states. A detailed analysis of white matter with DTI is possible owing to two of its features – mean diffusivity and fractional anisotropy (FA). Currently, the most widely used method of measuring anisotropy is DTI-FA which allows quantification, where the values obtained represent an average of the sampled fibers in a given region of interest (ROI). It is a highly sensitive but fairly nonspecific biomarker of neuropathology and microstructural architecture of white matter and is frequently considered a marker of white matter integrity.^30-31^ Several studies have demonstrated that the organization of white matter fiber bundles is the basis for DTI-FA. The myelin appears to influence its measurements, as does axonal integrity. The parallel organization of white matter fiber bundles is the basis for anisotropic diffusion, whereas myelin appears to modulate the amount of anisotropy.^30^ The analysis of ischemic lesions identified by neuroimaging and neuropathology shows reduced DTI-FA values, indicative of axonal damage and/or loss, and demyelination.^24,30-31^ However, the same analysis of regions visually identified as not affected, can also show derangement in the microarchitecture of the white matter, with axonal damage and demyelination.^32^ This appears to be the case of the midline corpus callosum. In spite of reports of atrophy and pathological confirmation of fiber loss, signal changes are rarely described in conventional neuroimaging.^3,13,16,17^

The objective of this study was to describe two segments of the corpus callosum, the genu and the splenium, in Binswanger’s disease patients using quantitative fractional anisotropy (DTI-FA), and compare results with a normal control group.

## Methods

The study included two samples, normal controls (n=12) and patients with Binswanger’s disease (n=12). The inclusion of BD patients was based on the National Institute of Neurological Disorders and Stroke and Association Internationale pour la Recherché etl’ Enseignement en Neurosciences (NINDS-AIREN) criteria,^33^ and assessment was performed with the Mini-Mental State Examination,^34^ Clinical Dementia Rating scale,^35^ and Hachinski ischemic score.^36^ The control subjects had no neuropsychiatric complaints, and presented results in the normal range following similar assessment. The characteristics of the subjects are displayed in Table 1.

**Table 1:** Characteristics of the sample.

	NC	BD
N	12	12
sex (m/f)	5/7	7/5
age (range)	74.8±5.1	77.6±8.6
education (years: m±sd)	12.4±2.43	9.67±4.56
NINDS-AIREN	negative	positive
MMSE^(a)^ (score: m±sd)	27.4±2.70	20.2±5.37
CDR^(b)^ (score)	0	1.50±0.64
Hachinski^(c)^ (score)	0.92±0.79	8.75±4.14
Fazekas^(d)^ (score)	2.0±0.85	6.0±0.0
leukoaraiosis (extension %)	--	≥75%

NC, normal controls; BD, Binswanger's disease; NINDS-AIREN, National Institute of Neurological Disorders and Stroke and Association Internationale pour la Recherché et l'Enseignement en Neurosciences (criteria for clinical diagnosis of vascular dementia);

(a) Mini Mental State Examination (short cognitive screening tool);

(b) CDR, Clinical Dementia Rating scale (global severity stages from 0 to 3);

(c) Hachinski, ischemic score (clinical assessment of vascular risk);

(d) Fazekas, white matter lesion scale (severity from 0 to 6).

### Techniques

**Figure 1:**
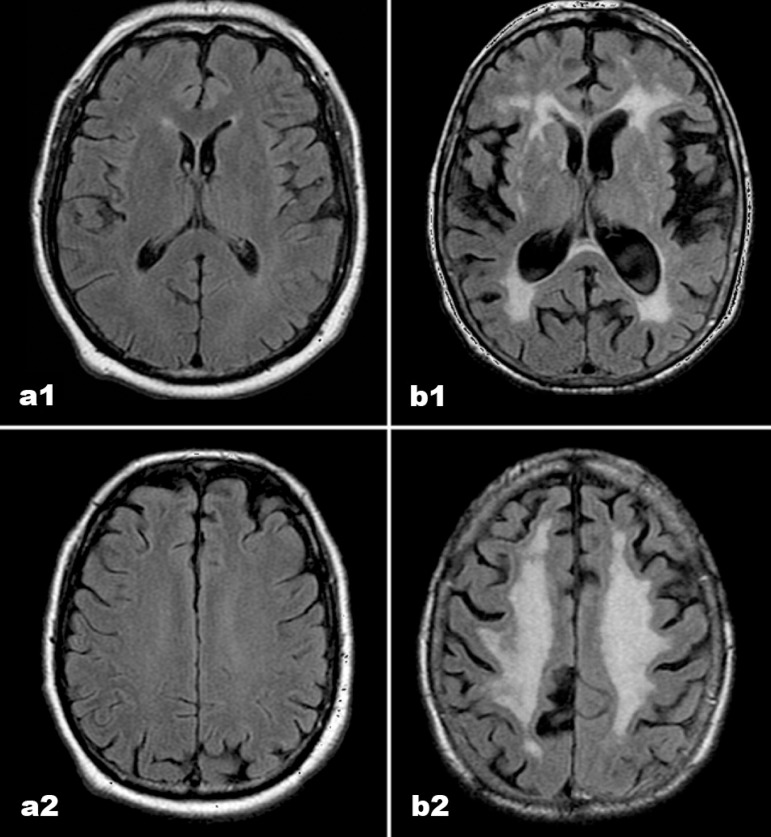
MR scans in FLAIR acquisition (axial plane) – sections at basal ganglia level (a1 and b1) and at supracallosal level (a2 and b2), in normal controls and Binswanger’s disease patients, respectively. The images represent examples from the study (Table 1).

A complete series of MR scans of the brain, with standard and DTI acquisitions, of the two samples was obtained using a 1.5T GE Signa Horizon machine. Axial plane fluid-attenuated inversion recovery (FLAIR) sequence scans were examined to evaluate the extension of the white matter lesions which were classified according to Fazekas’s scoring system^37-38^. The pathological cases had a Fazekas score=6 and LA≤75% (visual assessment), while the normal subjects yielded lower scores. The scoring was performed by two of the authors in consensus (DMM, EE) (Table 1 and Figure 1).

**Figure 2:**
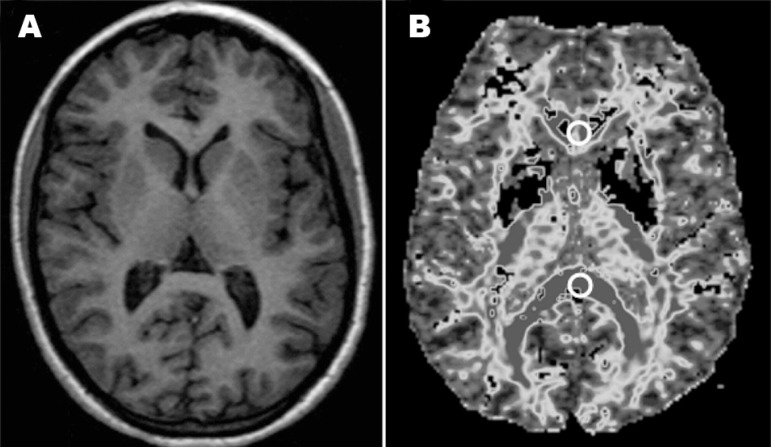
MR scans (axial plane) - sections at basal ganglia level: (A) 3DT1 sequence and (B) DTI-FA map. 3DT1 image for topo­graphical reference, DTI-FA map is shown to localize ROI placement.

The parameters used for DTI-FA acquisition correspond to those found in international studies on the subject, and are as follows: TR/TE=10000/89.1 msec, matrix=128×128, FOV=30×24 mm, NEX=1, b=1000 sec/mm^2^, slice thickness=5 mm, number of slices=30 without gap. Circular ROIs of 60mm^2^ were localized in the genu and the splenium of the corpus callosum on one axial plane parallel to the AC-PC line at the basal ganglia level of the DTI-FA maps (total number of ROIs=24 for each group) (Figure 2).

The DTI-FA maps were analyzed on an ADW 4.3 workstation using the Functool 4.5.3(GE Medical Systems). Statistical analysis (basic, ANOVA)^39^ was performed to compare intra-sample and inter-sample measures of the genu and splenium of the corpus callosum.

### Ethics

The present study is part of a larger project on Vascular Cognitive Disorder, approved by the Ethics Committee of IPUB-UFRJ. Informed consent was obtained from the participants or from a family member responsible, before study procedures.

## Results

The DTI-FA data of the genu and the splenium showed a significantly lower degree of anisotropy in Binswanger’s disease cases in comparison to normal controls (inter-sample). Considering each sample, there was also significant lower anisotropy measured between the genu and the splenium (intra-sample).

The obtained data and the significance among the regions are depicted in Table 2 and Table 3. Additionally the post-hoc Tukey HSD test was performed for improved representation of the results (Table 4).

**Table 2:** Results of quantitative FA in NC vs BD.

Regions	ROIs (n)	FA units (mean±sd)	
		NC	BD
Genu	12	0.6041±0.05	0.5555±0.08
Splenium	12	0.7230±0.04	0.6394±0.08
Total	24	0.6635±0.08	0.5975±0.09

**Table 3:** Statistical significance as shown with ANOVA.

ANOVA summary					
Source	SS	df	MS	F	p
Rows^(a)^	0.12	1	0.12	27.79	<0.0001
Columns^(b)^	0.05	1	0.05	11.58	0.0014
Rows × columns	0.01	1	0.01	2.32	0.1349
Error	0.19	44	0		
Total	0.37	47			

(a) inter-sample (genu and splenium - NC vs BD);

(b) intra-sample (genu vs splenium - NC and BD).

**Table 4:** Critical values for the Tukey HSD Test.

	HSD [0.05]	HSD [0.01]
Rows (2)	0.04	0.05
Columns (2)	0.04	0.05
Cells (4)	0.07	0.09

HSD, Highest significant difference.

## Discussion

The neuropathological characteristics of Binswanger’s disease are extensive subcortical white matter ischemic lesion, with axonal damage and myelin loss. The corpus callosum is also affected, frequently showing atrophy. These changes may be presently revealed by quantitative DTI-FA, an *in vivo* marker of fiber integrity. These white matter ischemic changes have been characterized with neuroimaging studies correlated with *post mortem* brain examination.^25,28^ The midline corpus callosum, but not its radiations, apparently suffers less in view of its rich vascular supply. The development of DTI-FA has provided a qualitative and quantitative evaluation of the white matter, and enabled assessment of the integrity of its constitutive fiber tracts.^30-31^

In spite of the infrequent description of signal changes on conventional neuroimaging,^3,17^ the DTI-FA shows that there may be a reduction of the measured values, indicative of fiber loss, related to axonal damage and demyelination, as revealed in neuropathological studies.^3,32,40^

The present data showed lower DTI-FA values of the two studied segments of the corpus callosum – genu and splenium – in BD in comparison to NC (inter-sample). There was also a differential change between the genu and the splenium in BD patients and in NC (intra-sample). The genu was more significantly affected in comparison to the splenium, suggesting an anterior-to-posterior gradient, as described previously.^41^

The literature on the issue is very scarce, and the bibliographical search yielded only a few related international studies. The published papers included data on the corpus callosum in Binswanger’s disease, compared to normal controls and to Alzheimer’s disease patients.^42-43^ In an earlier paper, the applied imaging technique was apparent diffusion coefficient (ADC) derived from the diffusion sequence, that represents the degree of diffusivity, in which ADC values and ratios (for the quantitative assessment of diffusion anisotropy) were calculated. ADCs in the corpus callosum (genu and splenium) were significantly higher in BD patients compared to controls, with disappearance of diffusion anisotropy, in a more significant way in the genu. These results suggest, according to the authors, that the cerebral white matter lesions in BD reflect a decrease in nerve fibers and diffuse myelin loss, and that the loss of nerve fibers in the corpus callosum may play a role in inducing cognitive decline.^42^

The results are comparable to those of the present study, even allowing for the differences between the techniques employed.

A more recent paper^43^ using the DTI-FA technique, presented an analysis of the corpus callosum of patients with vascular dementia (VaD) (included with criteria for Binswanger’s disease) in comparison to normal controls and Alzheimer’s disease patients.

The DTI-FA values of the corpus callosum (genu and splenium) in VaD were significantly lower in comparison to controls, and there were no statistically significant differences between genu and splenium of the corpus callosum in any group.

These results are in part comparable to those of the present study, as the inter-sample differences were statistically significant for both segments of the corpus callosum. In regard to the intra-sample differences between the genu and the splenium however, the present results were significant, in contrast to the cited study, where this difference may have been due to variation between the samples.

No papers on the subject were found in the national literature by the bibliographical search.

The corpus callosum, as the main neocortical commissure, establishes most of the inter-hemispheric connections and information transfer between areas related to cognition, behavior, motor and sensory functions.^2,6-9,44^ It participates in the large neural networks that support complex bi-hemispheric functions, and its damage may disrupt these networks and cause inter-hemispheric disconnection.

Disconnection of the anterior brain regions (prefrontal areas) due to genu damage, and of the posterior brain regions (mainly parieto-occipital areas) due to splenium damage may be related to impairment of high level inter-hemispheric integration,^2,6^ having a clear significant impact on the clinical performance of the patients.^40^ The interruption of connections of the genu fibers as well as cortical-prefrontal and subcortical-prefrontal fibers are of critical importance, where this multiple disconnection of the high-level prefrontal integrative regions may provide a structural basis for the impairment of the complex executive function cognitive domain, and a similar reasoning may be applied in relation to functions of parieto-occipital regions.^40-41,45-47^

Corpus callosum damage, together with that of the other white matter tracts seen in BD may, contribute to disconnection syndromes, one of the pathophysiological substracts of the VCI-VaD spectrum.^48-50^

## Conclusion

The corpus callosum frequently shows atrophy in Binswanger’s disease as a consequence of extensive centrum semiovale white matter ischemic lesion, with axonal damage and myelin loss. The changes of the corpus callosum may be currently revealed by quantitative DTI-FA, an *in vivo* marker of fiber integrity.

The studied regions of the corpus callosum of the brain of Binswanger’s disease patients, namely the genu (prefrontal interconnections) and splenium (parieto-occipital interconnections) showed lower DTI-FA values in comparison to normal controls. The genu is more severely compromised than the splenium. These results are indicative of loss of integrity of fibers that cross the corpus callosum, and suggest their interruption. Such findings represent an inter-hemispheric disconnection process, and compromise of the wide neural networks that are the basis of cognitive, behavioral, motor and sensory integration underlying the diverse clinical manifestations of the Binswanger’s disease subtype of the VCI/VaD continuum.
